# High levels of maternal total tri-iodothyronine, and low levels of fetal free L-thyroxine and total tri-iodothyronine, are associated with altered deiodinase expression and activity in placenta with gestational diabetes mellitus

**DOI:** 10.1371/journal.pone.0242743

**Published:** 2020-11-24

**Authors:** Sebastián Gutiérrez-Vega, Axel Armella, Daniela Mennickent, Marco Loyola, Ambart Covarrubias, Bernel Ortega-Contreras, Carlos Escudero, Marcelo Gonzalez, Martín Alcalá, María del Pilar Ramos, Marta Viana, Erica Castro, Andrea Leiva, Enrique Guzmán-Gutiérrez

**Affiliations:** 1 Laboratorio de Patologías del Embarazo, Departamento de Bioquímica Clínica e Inmunología, Facultad de Farmacia, Universidad de Concepción, Concepción, Chile; 2 Escuela de Tecnología Médica, Facultad de Ciencias de la Salud, Universidad San Sebastián, Chile; 3 Department of Medicine, Cedars-Sinai Medical Center, Los Angeles, CA, United States of America; 4 Group of Research and Innovation in Vascular Health (GRIVAS-Health), Chillán, Chile; 5 Vascular Physiology Laboratory, Department of Basic Sciences, Universidad del Bío-Bío, Chillán, Chile; 6 Laboratorio de Investigación Materno-Fetal (LIMaF), Departamento de Obstetricia y Ginecología, Facultad de Medicina, Universidad de Concepción, Concepción, Chile; 7 Facultad de Farmacia, Universidad CEU San Pablo, Ctra, Boadilla Km 5, Alcorcón, Madrid, Spain; 8 Departamento de Obstetricia y Puericultura, Facultad de Ciencias de la Salud, Universidad de Atacama, Atacama, Chile; University of Mississippi Medical Center, UNITED STATES

## Abstract

Gestational Diabetes Mellitus (GDM) is characterized by abnormal maternal D-glucose metabolism and altered insulin signaling. Dysregulation of thyroid hormones (TH) tri-iodethyronine (T_3_) and L-thyroxine (T_4_) Hormones had been associated with GDM, but the physiopathological meaning of these alterations is still unclear. Maternal TH cross the placenta through TH Transporters and their Deiodinases metabolize them to regulate fetal TH levels. Currently, the metabolism of TH in placentas with GDM is unknown, and there are no other studies that evaluate the fetal TH from pregnancies with GDM. Therefore, we evaluated the levels of maternal TH during pregnancy, and fetal TH at delivery, and the expression and activity of placental deiodinases from GDM pregnancies. Pregnant women were followed through pregnancy until delivery. We collected blood samples during 10–14, 24–28, and 36–40 weeks of gestation for measure Thyroid-stimulating hormone (TSH), Free T_4_ (FT_4_), Total T_4_ (TT_4_), and Total T_3_ (TT_3_) concentrations from Normal Glucose Tolerance (NGT) and GDM mothers. Moreover, we measure fetal TSH, FT_4_, TT_4_, and TT_3_ in total blood cord at the delivery. Also, we measured the placental expression of Deiodinases by RT-PCR, western-blotting, and immunohistochemistry. The activity of Deiodinases was estimated quantified rT_3_ and T_3_ using T_4_ as a substrate. Mothers with GDM showed higher levels of TT_3_ during all pregnancy, and an increased in TSH during second and third trimester, while lower concentrations of neonatal TT_4_, FT_4_, and TT_3_; and an increased TSH level in umbilical cord blood from GDM. Placentae from GDM mothers have a higher expression and activity of Deiodinase 3, but lower Deiodinase 2, than NGT mothers. In conclusion, GDM favors high levels of TT3 during all gestation in the mother, low levels in TT4, FT4 and TT3 at the delivery in neonates, and increases deiodinase 3, but reduce deiodinase 2 expression and activity in the placenta.

## Introduction

Gestational Diabetes Mellitus (GDM) is defined as any degree of glucose intolerance with onset or first manifestation during pregnancy [1]. GDM is characterized by abnormal maternal D-glucose metabolism and altered insulin signaling in feto-placental circulation [2]. It has been described that a decrease in maternal Free L-Thyroxine (FT_4_) levels during the first trimester of pregnancy could also increase GDM risk [3–5]. Several studies relate alterations in Thyroid Hormones (TH) levels with GDM, but their results are controversial. While some studies associate a decrease in maternal FT_4_ and increase in Thyroid Stimulating Hormone (TSH) with GDM, others have related an increase in maternal Total Tri-iodothyronine (TT_3_, Free T_3_ plus protein bound T_3_) with this pathology [6–8].

Throughout the gestational stage, the mother provides these hormones to the fetus through the placenta [9]. Fetal THs are fully contributed by the mother until week 16 of gestation after that, the fetus begins to produce its own THs, due to the maturation of the hypothalamus/pituitary/thyroid axis [10]. Few studies have evaluated the behavior of fetal THs in GDM. A decrease in the neonatal TH from mothers with type 2 diabetes mellitus was evidenced [11], while on the other hand, the suppression of these fetal hormones was also associated with maternal glucose intolerance [12]. However, there are no other studies that evaluate the neonatal thyroid profile from pregnancies with GDM. During pregnancy, T_4_ and T_3_ cross the placenta through Thyroid Hormone Transporters (THT), entering the placenta where they are regulated by deiodinases (DIO) [13, 14]. DIO are seleno-enzymes responsible for the catabolism of specific iodine atoms of the iodothyronine molecule [15–18]. These have three subtypes: I, II, and III (DIO1, DIO2, and DIO3, respectively), which have fundamental functions in thyroid hormone regulation [18, 19]. DIO1 is characterized by generating T_3_ through the deiodination of T_4_; however, its role is less relevant since its activity is lower than the other deiodinases [18]. DIO2 produces T_3_ from a 5′-deiodination of T_4_, increasing the availability of T_3_, which in turn is essential for the development of the fetus during pregnancy [20]. Indeed, it has been reported that a physiological concentration of thyroid hormones stimulates trophoblast endocrine function [21], while alterations in TH availability are linked with alterations in fetal growth and weight [22]. Finally, DIO3 is highly localized in placenta, and is the enzyme responsible for converting T_4_ into reverse T_3_ (rT_3_, inactive form), and catalyzes the conversion of diiodothyronine (T2) from T_3_, decreasing the levels of T_3_ [23, 24].

Currently, the metabolism of TH in placentas from pregnancies with GDM is unknown, then, we evaluated the levels of maternal and neonatal TH, and the expression and activity of placental DIO in the context of GDM.

## Materials and methods

### Patients, biological samples, and study groups

181 pregnant women were recruited between the years 2017 and 2018 from 3 primary health centers of Concepción (Víctor Manuel Fernández, Tucapel, and Santa Sabina) with 12–14 weeks of pregnancy. Pregnant women were followed through pregnancy until delivery. We collected blood samples during 10–14, 24–28, and 36–40 weeks of pregnancy, and total umbilical blood during post-delivery. Blood samples were transported to laboratory at 4°C. Sera were aliquoted and stored at -80°C. The placentae were obtained post-delivery, and transported to laboratory at 4°C. For the current study, a subset of 71 pregnant women who complied all diagnosis and inclusion criteria were analyzed.

Pregnant women between the 24–28 weeks of pregnancy with basal glycemia >90 mg/dL, (i.e., overnight starvation) or with >140 mg/dL at 2 h after an oral glucose load (75 g) were diagnosed as GDM, and subjected to dietary treatment with 1500 kcal/day and a maximum of 200 g per day carbohydrates. Our study obtained 23 pregnant with GDM and 48 pregnant without alteration in basal glycemia and oral glucose load, named Normal Glucose Tolerant (NGT). The exclusion criteria included underage women, previous diagnosis of diabetes mellitus and or thyroid pathologies, and other pregnancy pathologies (i.e. preeclampsia, fetal growth restriction, ectopic pregnancy).

The following characteristics were collected from the mothers: age (years), height (m), weight during first and third pregnancy trimesters (kg), and Body Mass Index (BMI) was calculated. For the newborn, data of gestational age (weeks), weight, height and ponderal index were collected.

#### Ethic aspects

The investigation conforms to the principles outlined in the Declaration of Helsinki. Ethics Committee approval from the Comité Ético Científico de Servicio de Salud Concepción (CEC:23-2017-20) and informed consent of each pregnant women from the three Primary Health Centers of Concepción were obtained.

### Clinical biochemistry analysis

Glucose was measured by a colorimetric assay (VITROS Immunodiagnostic Products, NY, USA, GLU Slides). TSH concentrations were measured by immunometric assay (VITROS Immunodiagnostic Products, NY, USA, TSH), while FT_4_, Total L-Thyroxine (TT_4_), and T T_3_ were measured by competitive immunoassay (VITROS Immunodiagnostic Products, NY, USA).

### Expression analysis

#### Reverse transcription and quantitative RT-PCR

RNA was extracted using Trizol reagent (Life Technologies Corporation, Carlsbad, CA, USA) following the manufacturer's instructions. Reverse transcriptions were realized using ImProm-II™ Reverse Transcription System (Promega, Madison, WI, USA). 1 μg of RNA in presence of 0.5 μg of random primers mix is thermally denatured (70°C, 5 min) and cooled on ice. A reverse transcription reaction mix was assembled on ice to contain nuclease-free water (to a final volume of 15μl), reaction buffer 5x, 160UI of reverse transcriptase, 6mM of magnesium chloride, 0.5 mM of dNTPs, 20UI of ribonuclease inhibitor and 1UI of Recombinant RNasin® Ribonuclease Inhibitor (Promega). As a final step, the template-primer combination is added to the reaction mix. Following an initial annealing (25°C, 5 min), and the reaction is incubated at 42°C for up to one hour. This procedure outlines the method proposed to amplify the entire 20μl reaction.

cDNA amplifications were performed using a Step One real time PCR system (Applied Biosystem, CA, USA) in a reaction mix containing 0.2 μM primers and master mix provided in the brilliant SYBR green qPCR Master Mix (Applied Biosystem, CA, USA) as described [25]. Hot Start Taq DNA polymerase was activated (10 min, 94°C), and assays included a 94°C denaturation (30 s), annealing (30 s) at 54.0°C (DIO3 and 28S) or 57.2°C (DIO1 and DIO2), and extension at 72°C (DIO1, DIO2, DIO3 and 28S, 45 s). Fluorescent product was detected after 3-s step to 5°C below the product melting temperature (Tm). Product specificity was confirmed by agarose gel electrophoresis (2% w/v) and melting curve analysis. The product Tm values were 60°C for DIO1, 60.2°C for DIO2, 56.8°C for DIO3 and 86.7°C for 28S [25]. Oligonucleotide primers are as follows: DIO1 (sense) 5’ -AGCGACTAGAGGACACGACT- 3’, DIO1 (anti-sense) 5’ -ACCAGTGGCCTATTACCTTGC- 3’, DIO2 (sense) 5’ -TCGATGCCTACAAACAGGTGAA- 3’, DIO2 (anti-sense) 5’ -TTGCCACTGTTGTCACCTCC- 3’, DIO3 (sense) 5’ -TCGAGCGTCTCTATGTCATC- 3’, DIO3 (anti-sense) 5’ -TCATCATAGCGTTCCAACCA- 3’, 28S (sense) 5’ -TTGAAAATCCGGGGGAGAG- 3’, 28S (anti-sense) 5’ -ACATTGTTCCAACATGCCAG- 3’. Expected size products for DIO1 (115 bp), DIO2 (94 bp), DIO3 (109 bp) and 28S (100 bp) were confirmed in PCR experiments. The 28S rRNA ct was unaltered (P > 0.05, n  =  16) in all experimental conditions (not shown). The Ct value was defined as the PCR cycle number at which the probes’ fluorescent signal exceeded the background signal and was used to calculate the gene-expression data with the 2^–ΔΔCt^ method.

#### Western blotting

Proteins were extracted taking 1 mg of placenta in presence of lysis buffer (Tris 50mM pH 7.5; Triton X-100 0.2%; EDTA 1mM), and proteases inhibitor (Halt^TM^ Protease cocktail, Thermo Fisher Scientific, Waltham, MA, USA)

Proteins (70 μg) separated by polyacrylamide gel (12%) electrophoresis were probed with a primary polyclonal rabbit anti-DIO2 (1:1000, v/v) (Thermo Fisher Scientific), rabbit anti-DIO3 (1:1000, v/v) (Thermo Fisher Scientific), or monoclonal mouse anti-β-actin (1:3000, v/v) (Santa Cruz Biotechnology, Santa Cruz, CA, USA) antibodies. Proteins were detected by enhanced chemiluminescence in a LI-COR C-DiGit™ Blot Scanner 3600 (Lincoln, NE, USA) and quantified by densitometry using software ImageJ (NIH, Bethesda, MD, USA).

### Placental tissue analysis

#### Immunohistochemistry

A standard protocol for immunohistochemistry was applied to two sections of the same placenta sample (100mm^3^) to study each DIO. The tissue sections were mounted on a slide previously treated with 2% silane in acetone. The placenta sections were dewaxed with three xylol baths of 10 minutes each and then were rehydrated in an ethanol battery (absolute ethanol, 95%, 70%, respectively) of 1 minute each. Afterwards, an antigenic recovery was performed with 0.01M Sodium Citrate buffer solution pH 6.0 at a temperature of approximately 90°C for 30 minutes, using a steamer.

The samples were then cooled in the same solution with changes of distilled water. A wet chamber was needed for the following steps. The endogenous peroxidase was blocked using 3% hydrogen peroxide in absolute methanol for 10 minutes at room temperature, followed by a wash with 1X PBS Buffer at pH 7, 0.01M + Tween 20 0.05% (PBST). The samples were then incubated with the respective primary antibody at DIO1 1:50 (v/v), DIO2 1:100 (v/v), and DIO3 1:500 (v/v) concentrations with TBS antibody diluent, for 45 minutes at 37°C, followed by washing with PBST.

A detection polymer (Mouse / Rabbit PolyDetector Plus DAB HRP Brown Detection System Bio SB) was used for to detect the primary antibody, incubating each sample for 15 minutes at 37°C and subsequently washed with PBST. Finally, a nuclear contrast was carried out with Harris's Hematoxylin for 20 seconds, followed by a bluish in running water for 5 minutes, then by a dehydration battery of increasing ethanol (70%, 95%, absolute ethanol) of 1 minute each, to end with a xylol clearance for 3 minutes and the assembly of the slides with synthetic resin in hydrophobic medium.

### Deiodinase activity

300 μg of placenta protein from cotyledon in presence of 1,4-Dithiothreitol (DTT, 20mM) were exposed to T_4_ (MP Biomedicals, LLC, Solon, OH, USA) (0–500 nM, 37°C, 15 min). The reaction was stopped with absolute ethanol (4°C). Then, the sample was centrifuged at 10.500g (4°C, 8 min), and the supernatant was used to measure T_3_ and rT_3_ [26, 27].

#### T_3_ and rT_3_ measure

T_3_ (Competitive ELISA, Invitrogen) and rT_3_ (Competitive ELISA, Invitrogen) were quantified from supernatants.

#### Determination of kinetic parameters

Saturable T_4_ conversion at initial rates was adjusted to the Michaelis-Menten asymptotic hyperbola. T_4_-conversion kinetic parameters maximal velocity (Vmax) and apparent Michaelis-Menten constant (Km) of transport were calculated as follows:

$$Vo=\frac{Vmax•[T_{4}]}{Km+[T_{4}]}$$ (1)

Where Vo correspond to initial velocity and [T_4_] the T_4_ concentration. Each assay was run in duplicate and activity was expressed in ng/mL/μg of protein/min (5’-deiodinase activity) and pg/L/μg of protein/min (5-deiodinase activity). To compare the GDM effect on deiodinase activity, we calculated the enzyme catalytic efficiency using the Vmax/Km ratio.

### Statistical analysis

Sample size was determined by average comparison approach considering free T4 level reported [28] in Chilean pregnant women with the equation:

$$n=\frac{2{\left(Z_{\alpha }+Z_{\beta }\right)}^{2}\;x\;S^{2}}{d^{2}}$$ (2)

Replacing free T4 level during first trimester of pregnancy (S), Z_α_ value of 0.05, Z_β_ value of 0.8 and d value of 0.5; the estimated sample size (n) per group was 70 pregnant women. Thus, this study required an estimated population of 140 pregnant women. Values are mean ± standard deviation (S.D.) (or range or SEM), with n  =  71 different parameters from NGT or GDM pregnant women and their newborns. The normality of the data was calculated with Kolmogorov-Smirnov test. Comparisons between two and more groups were performed by means of Student’s unpaired t test and analysis of variance (ANOVA), respectively. If the ANOVA demonstrated a significant interaction between variables, post hoc analyses were performed by the multiple-comparison Bonferroni correction test. P < 0.05 was considered statistically significant. The statistical software GraphPad Prism 7.0a.65 (GraphPad Software Inc., San Diego, CA, USA) was used for data analysis.

## Results and discussion

### Maternal thyroid hormone profile is altered in GDM during pregnancy

Our study considered the following along pregnancy of 71 women with either NGT or GDM pregnancies. No significant changes were found in height, overall weight during first trimester and third trimester, and BMI between NGT and GDM. However, we observed an increased in weight gain in GDM with respect to NGT. According to diagnosis criteria, increases in basal glycaemia, glycaemia 2 hours post 75 grams of glucose were found during second trimester of GDM pregnancies compared with NGT pregnancies (Table 1).

**Table 1:** Maternal clinical and biochemical parameters in NGT and GDM pregnancies.

Parameters	Units	NGT	GDM
**n**		48	23
**age**	years	29.9 ± 5.6 (27.9–31.9)	29.5 ± 6.9 (26.0–33.1)
**Height**	M	1.60 ± 0.1 (1.57–1.61)	1.61 ± 0.1 (1.55–1.66)
**Weight 1T**	kg	77.8 ± 5.6 (75.3–80.1)	78.3 ± 5.2 (75.2–80.3)
**Weight 3T**	kg	88.8 ± 9.3 (85.5–91.1)	91.5 ± 5.5 (88.2–93.7)
**Weight Gain (3T-1T)**	kg	11.0 ± 1.0 (9.9–11.8)	13.2 ± 1.9* (12.3–14.1)
**BMI 1T**	kg/m^2^	30.3 ± 3.9 (28.5–32.0)	30.2 ± 3.8 (28.1–32.3)
**BMI 3T**	kg/m^2^	32.3 ± 5.6 (29.1–35.5)	31.5 ± 4.0 (29.5–33.4)
**Basal Glycaemia 1T**	mg/dl	81.1 ± 5.5 (78.7–83.7)	81.6 ± 2.1 (80.7–82.9)
**Basal Glycaemia 2T**	mg/dl	82.4 ± 0.9 (81.9–82.8)	87.7 ± 4.8* (85.9–90.1)
**Glycaemia 2h post 75 g (2T)**	mg/dl	109.0 ± 9.6 (101.2–114.4)	161.3 ± 9.7* (155.8–172.4)
*Maternal thyroid hormone profile at the first trimester*			
**TSH**	mIU/L	2.0 ± 1.3 (1.7–2.6)	2.3 ± 1.9 (1.2–3.4)
**FT**_**4**_	ng/dl	1.0 ± 0.2 (0.9–1.1)	0.9 ± 0.2 (0.8–1.0)
**TT**_**4**_	μg/ml	11.4 ± 1.9 (10.5–12.4)	11.5 ± 1.7 (10.7–12.5)
**TT**_**3**_	ng/ml	1.7 ± 0.2 (1.6–1.8)	1.9 ± 0.1* (1.9–2.0)
*Maternal thyroid hormone profile at the second trimester*			
**TSH**	mIU/L	2.0 ± 0.8 (1.6–2.4)	2.9 ± 1.2* (2.3–3.5)
**FT**_**4**_	ng/dl	0.8 ± 0.1 (0.7–0.9)	0.8 ± 0.3 (0.6–0.9)
**TT**_**4**_	μg/ml	12.7 ± 1.8 (11.6–13.4)	13.3 ± 3.0 (11.8–14.9)
**TT**_**3**_	ng/ml	1.9 ± 0.3 (1.7–2.0)	2.2 ± 0.2* (2.1–2.3)
*Maternal thyroid hormone profile at the third trimester*			
**TSH**	mIU/L	2.1 ± 0.4 (1.9–2.3)	3.4 ± 1.0* (2.9–3.9)
**FT**_**4**_	ng/dl	0.7 ± 0.1 (0.6–0.8)	0.7 ± 0.1 (0.6–0.7)
**TT**_**4**_	μg/ml	11.1 ± 1.7 (10.1–12.3)	12.5 ± 1.4* (11.7–13.3)
**TT**_**3**_	ng/ml	1.6 ± 0.2 (1.5–1.7)	2.3 ± 0.1*† (2.2–2.3)

NGT: Normal tolerance glucose. GDM: gestational diabetes mellitus.1T: first trimester; 2T: second trimester; 3T: Third trimester; BMI: Body Mass Index. TSH: Thyroid Stimulant Hormone. FT_4_: Free Thyroxine. TT_4_: Total Thyroxine. TT_3_: Total triiodothyronine. Mean ± S.D. (range).

*P<0.05 versus NGT.

†P<0.05 versus TT_3_ at the first trimester.

We analyzed the thyroid hormone profile (i.e., TSH, FT_4_, TT_4_ and TT_3_) during pregnancy, in which FT_4_ levels were similar in both groups along pregnancy. However, women with GDM showed an increase in the TT_3_ levels in all three trimesters of pregnancy compared to NGT. In addition, TT4 levels was higher in third trimester, and TSH levels were higher in GDM at second and third trimester than respective value in NGT. TT_3_ levels at the third trimester were higher than levels measured at first trimester only in the GDM group. Similar results have been described by other authors. In a HAPO study with 600 participant women, TT_3_ levels were increased in GDM pregnant during second trimester, including a positive correlation with glycaemia at 2 hours post-glucose 75 grams [29]; however, no changes were observed for TSH levels. Another study measured Free T_3_ (FT_3_), observing an increase of this analyte during first and second trimesters; although FT_3_ levels during the third trimester were not evaluated in this study [8]. In a retrospective cohort study in Israel with singleton pregnant women who had a first trimester and at least one additional (either first, second, third trimesters, or after delivery) TSH measurement for the same pregnancy, low TSH levels during first trimester were correlated with lower rates of GDM [30]. Moreover, a relationship between low FT_4_ levels and high maternal weight during second trimester was associated with an increased GDM risk; however, T_3_ was not measured [2]. In the other hand, another study in Pakistan observed that GDM women had high TSH blood levels, which were correlated with poor glycemic control and BMI [22]. Interestingly, a similar condition known as Gestational Impaired Glucose Tolerant (GIGT), which is associated with glucose at 2 hours post-75 grams > 100mg/dl, but <140 mg/dl, shown an increase in FT_4_ and TT_4_ compared to controls, without changes for TT_3_ [12], which could indicate that a TT_3_ increase could be associated with GDM, but not with other diseases.

#### Thyroid hormone profile is altered in newborn from GDM pregnancies

Newborn clinical parameters did not show any change associated with height, ponderal index and sex proportion (Table 2). However, gestational age was reduced; and weight was increased in GDM pregnancies. In addition, the thyroid hormone profile obtained from umbilical cord blood from GDM pregnancies showed a reduction in TT4 (~13.2%) and FT_4_ (~25%) and TT_3_ (~47%) levels, and an increase in TSH levels compared with newborn from NGT pregnancies.

**Table 2:** Newborn clinical and biochemical parameters in NGT and GDM pregnancies.

Parameters	Units	NGT	GDM
**n**		48	23
*Newborn characteristic*			
**Gestational age**	weeks	38.1 ± 1.1 (38.4–38.7)	37.3 ± 1.4* (36.5–38.0)
**Weight**	g	3600 ± 350 (3450–3740)	3900 ± 320* (3760–4050)
**Height**	cm	50 ± 3 (48.5–51.5)	51 ± 3 (49.5–52.5)
**Ponderal index**	g/cm^3^	2.88 ± 0.43 (2.66–3.02)	2.94 ± 0.26 (2.81–3.07)
**Sex**	Female/Male	23/25	11/12
*Thyroid hormone profile (umbilical cord blood)*			
**TSH**	mIU/L	3.2 ± 0.5 (2.9–3.5)	3.8 ± 0.8* (3.4–4.2)
**FT**_**4**_	ng/dl	1.2 ± 0.1 (1.1–1.2)	0.9 ± 0.1* (0.8–0.9)
**TT**_**4**_	ug/ml	12.1 ± 1.4 (11.4–12.8)	10.5 ± 1.8* (9.4–11.3)
**TT**_**3**_	ng/ml	1.9 ± 0.2 (1.8–2.0)	1.0 ± 0.1* (0.9–1.0)

NGT: Normal glucose tolerance; GDM: gestational diabetes mellitus; TSH: Thyroid Stimulant Hormone; FT_4_: Free Thyroxine: TT_4_: Total Thyroxine; TT_3_: Total triiodothyronine. Data correspond to mean ± S.D. (range).

*P<0.05 with respect to NGT.

There are few studies where TT_4_, FT_4_ or TT_3_ have been measured from GDM umbilical cord blood. A retrospective study with diet-treated GDM mothers, TSH levels in umbilical venous blood obtained at delivery was progressively increased with the severity of glucose intolerance in the mother, while FT_4_ levels in the fetus were normal, proposing the high TSH levels as a response to fetal hypoxic stress, rather than as a response to GDM [31]. Another study later observed a reduction in fetal TT_4_ and TT_3_ levels, and an increase in fetal TSH levels in conditions of GIGT with respect to control pregnancies [12]. These alterations were associated with a thyroid unbalance in the fetus. With this information is possible to suppose that placenta could play a role in the reduction of T_3_ and T_4_ levels in fetal blood via DIOs.

#### Deiodinase expression is altered in placenta from GDM pregnancies

**Fig 1:**
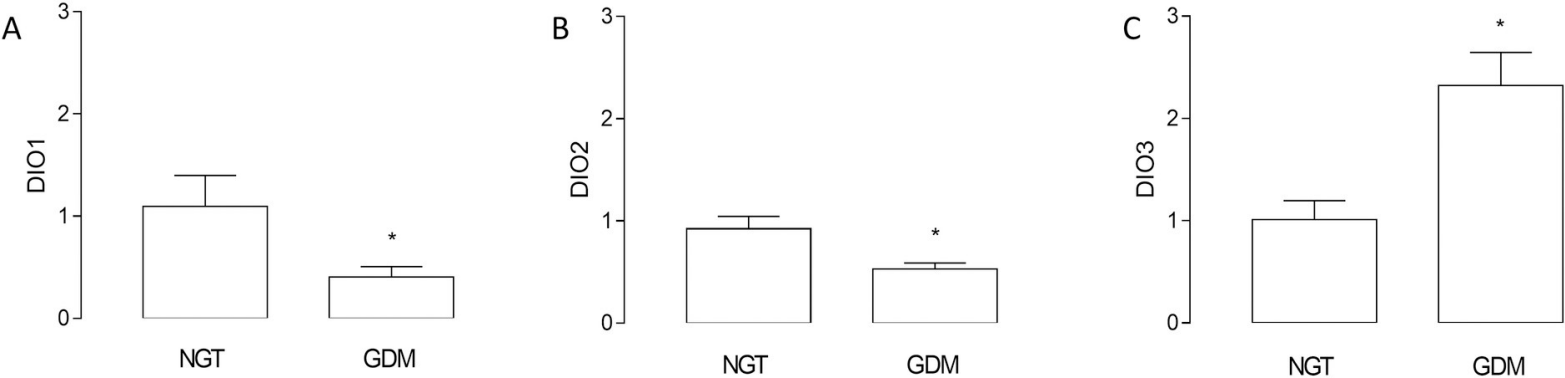
GDM effect on mRNA levels for deiodinases in human placenta. Deiodinases (DIO1, DIO2, and DIO3) mRNA levels were measured using Q-RT-PCR (2^-ΔΔCT^) in placentas from gestational diabetes mellitus (GDM) and normal glucose tolerance (NGT) pregnancies. Values are mean ± SE. *P<0.05 compared to NGT pregnancies. n = 20 per group.

**Fig 2:**
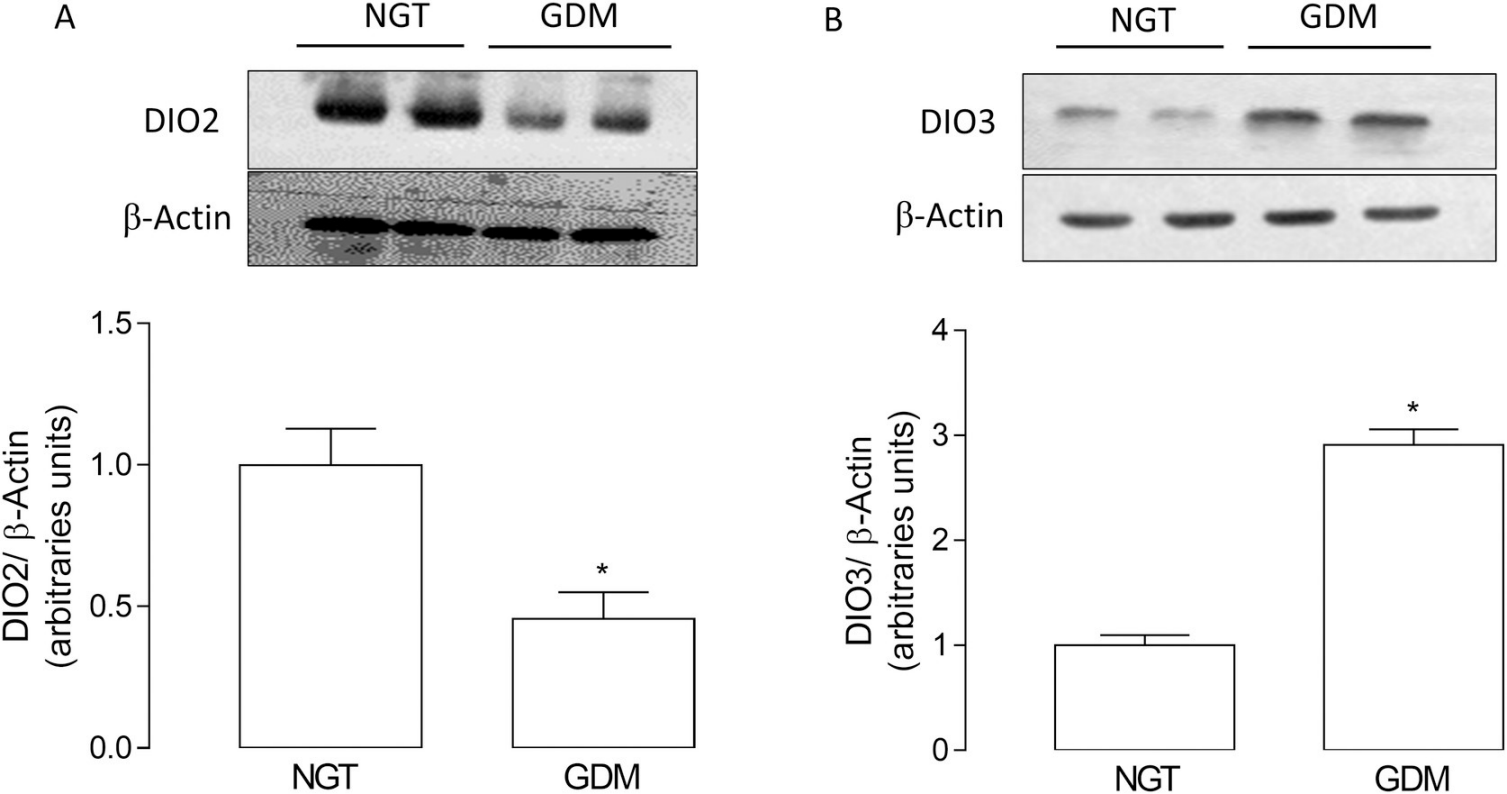
GDM effect on protein levels of deiodinase in placenta. Deiodinase (DIO) protein levels were measured using western blot in placentae from normal glucose tolerant (NGT) and gestational diabetes mellitus (GDM) pregnancies. (A) DIO2 and (B) DIO3 protein levels in placentae from GDM and NGT pregnancies. β-actin was used as a load control. Results of densitometric analysis were expressed as DIO/β-actin. Values are mean ± SE. *P<0.05 compared to NGT. n = 8–9 per group.

We found a reduction (~47%) in the mRNA levels of DIO2; but an increase (~2.3 fold) in the mRNA levels of DIO3 in GDM compared to NGT pregnancies. mRNA levels of DIO1 were similar in both conditions (Fig 1). Accordingly, protein levels of DIO2 were reduced (~54%), but DIO3 was increased (~2.9 fold) in placenta from GDM pregnancies (Fig 2). Furthermore, it was not possible to determine DIO1 protein levels with this technique (not shown), as it has been evidenced in literature that DIO1 protein is not found in placenta [24, 32].

**Fig 3:**
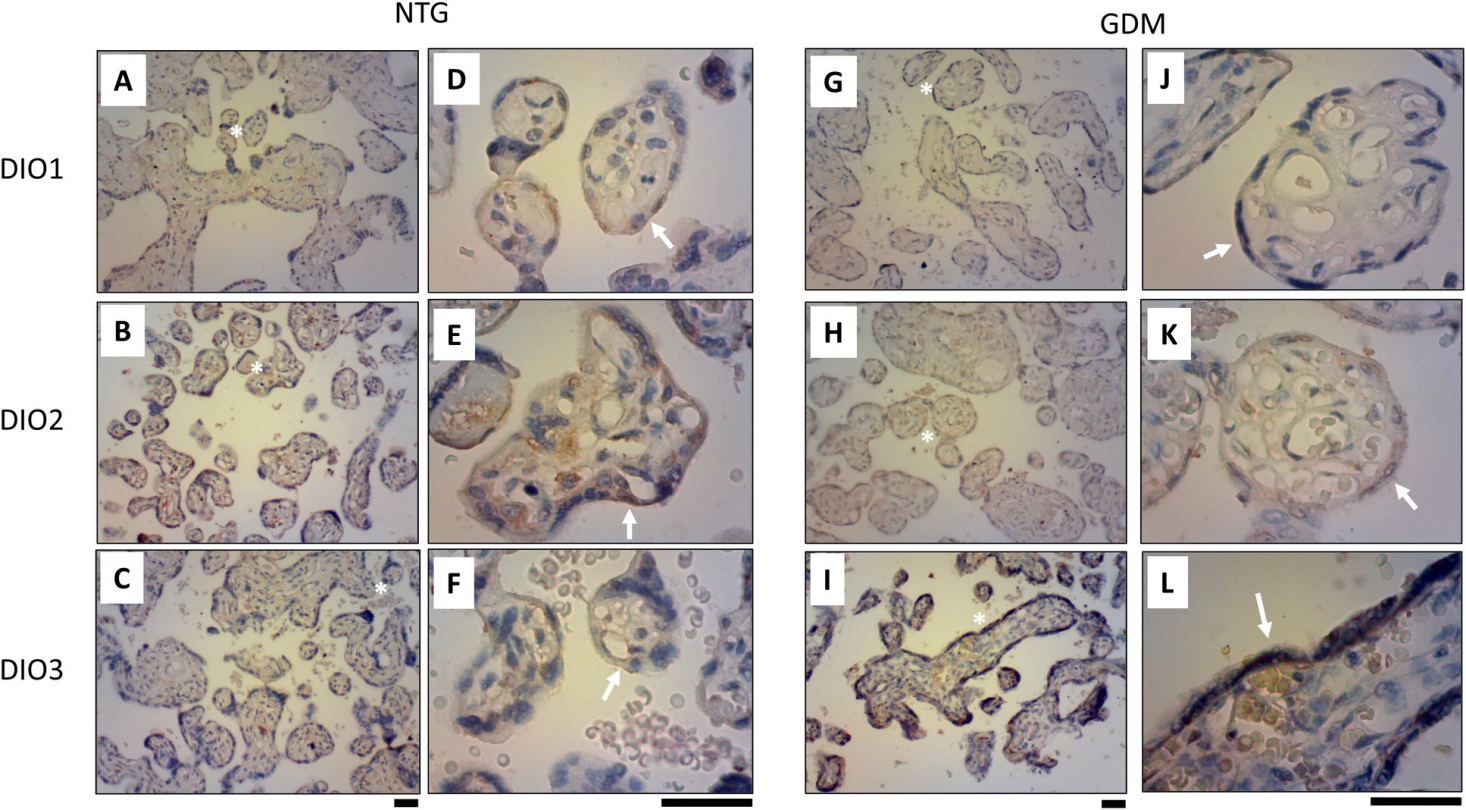
GDM effect on DIO2 and DIO 3 expression in placental tissue. DIO2 and DIO3 immunodetection were realized by immunohistochemistry (IHC) in normal glucose tolerant (NTG, A-F) and gestational diabetes mellitus (GDM, G-L) pregnancies. In A-C and G-I 400x magnification, and in D-F and J-L 1000x magnification. White asterisk (*) in A-C and G-I indicates a magnification point in D-F and J-L, respectively. White arrow in D-F and J-L indicates syncytiotrophoblast in the placental tissue. Black bar: 50μm. N = 5 per group.

To determine the expression and location of DIO2 and DIO3 proteins in the placenta, we used IHC. As mentioned above, we did not find any changes for DIO1 in placental tissue from NTG (Fig 3A–3D) and GDM (Fig 3G and 3H), which could be unspecified marker. Was determined that DIO2 and DIO3 are expressed mainly in the syncytiotrophoblast (Fig 3). Confirming previous results using Q-PCR and western blot, DIO2 had less immunoreactivity in GDM placentae (Fig 3H–3K) compared to NGT (Fig 3B–3E), whereas DIO3 had a major immunoreactivity in GDM (Fig 3I–3L) compared to NGT (Fig 3C–3F) placentae.

In this regard, other authors have described the presence of deiodinases in the placenta. Thus, mRNA expression levels of DIO2 were significantly higher in normal placenta during the first 20 weeks of pregnancy compared with term placenta, while in the other hand, DIO3 was significantly increased throughout gestation compared with term samples [33]. During the first trimester, the syncytiotrophoblast from normal placenta showed strong DIO3 and weak DIO2 immunoreactivities, while stronger DIO2 immunoreactivity compared to DIO3 during the third trimester was found [23, 33]. These results suggest that the higher expression of DIO3 may regulate T_3_ levels crossing from the mother to the fetus.

Currently, some studies have evidenced opposite changes in DIO2 and DIO3 in other pathologies. An increase in DIO2 and a decrease in DIO3 mRNA expression in the hippocampus, amygdala, and prefrontal cortex have been evidenced in mice with epilepsy [34]. On the other hand, in papillary thyroid tumors, it has been shown that the expression of DIO2 was reduced and DIO3 was increased, by the ERK signaling pathway [35]. Likewise, it has been observed that in an oxygen-glucose deprivation/reperfusion model (OGDR) of a human cardiomyocyte cell line (AC16, HCM), there is a decrease in the mRNA and protein levels of DIO2 and increase of DIO3 [36], which are similar results to those found in preterm infants coursing with intraventricular hemorrhage, where the authors suggest a potential mechanism mediated by hypoxia-ischemia and oxidative stress [37]. These findings are associated with studies in cerebral ischemia in rat [38], and oxidative stress in culture rat astrocytes [39]. All these changes suggest that these enzymes possibly are mediated by a competitive mechanism dependent of hypoxia and oxidative stress, which are conditions present in GDM as well [40, 41].

While no changes in deiodinases have been observed in immunoreactivity for other pregnancy pathologies such as preeclampsia [42], this is the first evidence showing significant difference in immunoreactivity between normal and GDM placenta for both DIO2 and DIO3.

#### Deiodinase activity is altered in placenta from GDM pregnancies

**Fig 4:**
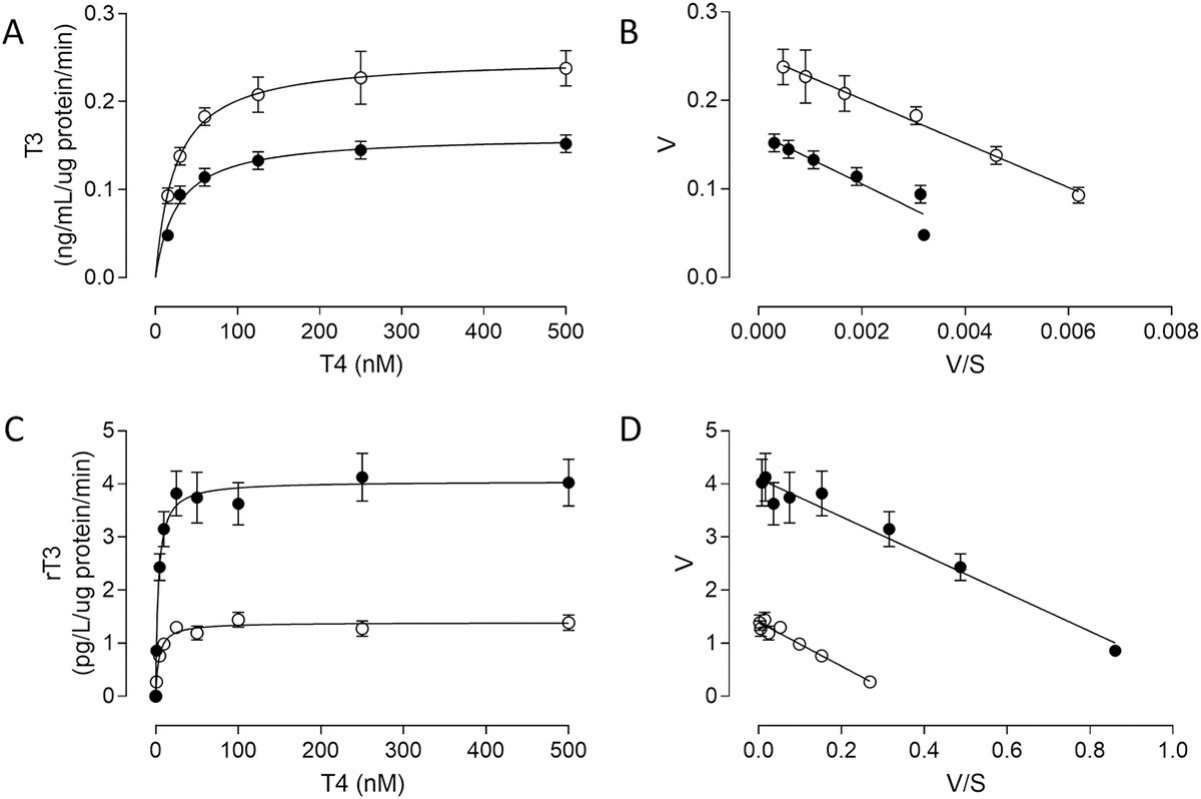
GDM effect on 5’- and 5- deiodinase activity in human placenta. Kinetic of deiodinase activity was measured as T_3_ or rT_3_ formation from T_4_ (0–500 nM, 37°C, 15 min) in placental tissue (300μg placental protein) from Normal Glucose Tolerant (NGT, ○) and Gestational Diabetes Mellitus (GDM, ●) pregnancies. In A and C, T_3_ and rT_3_ formation from T_4_ adjusted to Michaelis-Menten equation. In B and D, Eadie-Hofstee plot from data in A and C, respectively. Mean ± SEM. N = 9 per group.

DIO activity is presented as kinetic enzymatic assays (Fig 4) and parameters (Table 3). We observed that T_3_ and rT_3_ formation from T_4_ were saturable in placentae from NGT and GDM pregnancies (Fig 4A–4C). Eadie-Hofstee plots were lineal in both groups of placentae. 5’deiodinase activity showed a reduction in *V*_max_ (~35%), without changes in apparent *K*_m_ in placentae from GDM compared to NGT (Table 3). However, the 5-deiodinase activity showed an increase in *V*_max_ (~2.9 fold), without changes in apparent *K*_m_ in GDM compared with NGT (Table 3).

**Table 3:** Kinetic parameters of 5- and 5’- deiodinase activity.

Kinetic parameters	NGT	GDM
***5-deiodinase activity (T***_***4***_ ***→ rT***_***3***_***)***		
***V***_**max**_ (pg/L/μg of protein/min)	1.39 ± 0.04	4.10 ± 0.09 *
***K***_**m**_ (nM)	4.14 ± 0.36	3.59 ± 0.27
***V***_**max**_**/*K***_**m**_ (pg/L/μg of protein/min/nM)	0.33 ± 0.03	1.14 ± 0.10*
***5’-deiodinase activity (T***_***4***_ ***→ T***_***3***_***)***		
***V***_**max**_ (ng/mL/μg of protein/min)	0.251 ± 0.003	0.163 ± 0.012*
***K***_**m**_ (nM)	24.92 ± 0.91	28.61 ± 5.64
***V***_**max**_**/*K***_**m**_ (ng/mL/μg of protein/min/nM)	0.010 ± 0.001	0.006 ± 0.002*

Vmax: maximal velocity; Km: Michaelis-Menten constant; Vmax/Km: enzyme catalytic efficiency.

*P<0.05 with respect to NGT. N = 5 per group.

Deiodinase activity is classified in two types: (1) 5-deiodinase, favoring T_3_ formation from T_4_ mediated by DIO1 and DIO3, and (2) 5’-deiodinase, favoring rT_3_ formation from T_4_, or T_2_ from T_3_, mediated by DIO1 and DIO2.

An inverse relationship between FT_4_ and GDM has been confirmed, identifying a prevalence of GDM of 17.25% in the lowest FT_4_ quintile, decreasing to 11.62% in the highest FT_4_ quintile [6], which suggests changes in deiodinase activity in this type of patients. Another study confirmed the previous speculation that the T_3_/FT_4_ ratio (a measure of deiodinase activity) is related to maternal weight and glucose if weight-driven deiodinase activity may contribute to glycemic activity through T_3_ stimulation [29, 43].

The Km values that we obtained from 5-deiodinase activity are similar to those from previous experiments with that have measured DIO3 activity in placental tissue [44, 45], however, our Km values for 5’-deiodinase activity show less affinity for T_4_ than those that have been previously described for DIO2 activity in placental tissue [26, 46]. The reason for this could be that the methodology used (immunoassay versus radioactivity assay) the affinity for DIO2 in an unknown way, or that there is another, currently unknown enzyme or factor that affects it.

Studies in normal placenta have shown higher activity of DIO3 than DIO in first trimester and term placenta [33], while fetuses showed significantly lower TT_3_, but higher rT_3_ levels than newborns [47]. These results might be related with the DIO2 and DIO3 activity observed in the placenta. These changes in deiodinase activity have been associated with oxidative stress, specifically an increase in DIO3 activity and a decrease in DIO2 activity [39]. Our findings showing a change of deiodinases expression and activity in GDM placenta highlight the hypothesis that thyroid dysregulation occurs in the GDM fetus and that the required T_3_ for the fetus would not be optimal.

There were certain limitations in our study. Initially, the sample size was lower than total number of pregnant women recruited, due to miscarriage, suffering another, non-notified pregnancy pathology, or some of them no longer wanted to participate. Moreover, as we used term placentae, all the parameters analyzed are a reflect of the end of pregnancy and might not be accurate as a portrayal of the pregnancy course, specially of the first trimester.

## Conclusions

**Fig 5:**
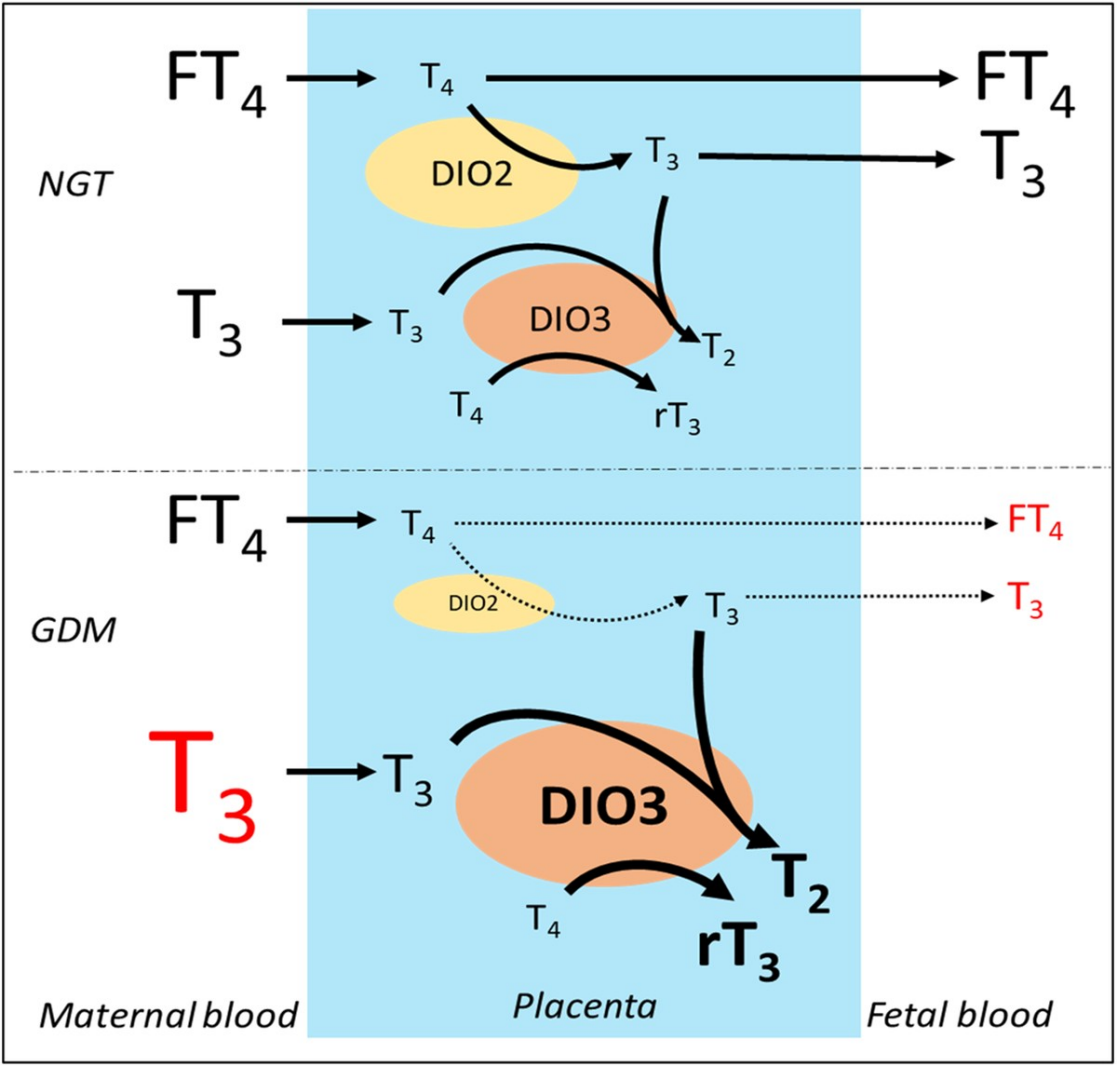
Proposal model for placental deiodinases in gestational diabetes mellitus (GDM) mothers. In normal glucose tolerant pregnancies (NGT), the thyroid hormones Free T_4_ (FT_4_) and T_3_ are transported from the maternal blood through the placenta, where their concentrations are regulated by deiodinase 2 (DIO2), which converts T_4_ to T_3_; and deiodinase 3 (DIO3), which converts T_3_ to T_2_, and T_4_ to rT_3_; in order to keep a steady and healthy concentration of thyroid hormones from the mother to the fetus. In GDM however, in order to prevent an influx of an excess of T_3_ from the mother to the fetus, the expression and activity of DIO3 increases while DIO2 decreases, causing less T_3_ to be transported to fetal blood by a currently unknown mechanism.

An adequate concentration of THs is essential for the normal development of the fetus during pregnancy. At early stages, the mother provides THs, which are transported and regulated through the placenta before being released to the fetal blood. Deiodinases are responsible for the fine regulation of T_3_ and T_4_ that will be finally transported to the fetus, by inactivating T_4_ to rT_3_ (DIO2) or by converting T_3_ to T_2_ (DIO3), both rT_3_ and T_2_ having no apparent genomic biological functions. GDM mothers have higher levels of TT_3_, while expression and activity of DIOs in placenta are altered as well, as summarized in Fig 5. This leads to thyroid dysfunctions in the fetus even at early stages of the pregnancy. The higher levels of TT_3_ present in GDM mothers, regardless of the pregnancy trimester, are not reflected in the fetal blood, where both FT_4_ and TT_3_ are reduced in comparison to the mother. This could be explained as an altered compensatory mechanism to prevent an excess of T_3_ in the fetus, as any alteration could lead to an impaired development of the fetus [48, 49]. In conclusion, TH levels in GDM mothers are higher than those of normal mothers, peaking at the third trimester for both TT3 and TSH. The opposite was observed in the newborn, having decreased levels of FT_4_ and TT_3_ in the umbilical cord blood. This is associated with alteration of expression and activity of placental DIOs, observing a reduction of DIO2 and an increase of DIO3 in GDM pregnancies. The mechanism behind these alterations was not studied in this work, but it would be likely mediated by the hypoxia and oxidative stress present in GDM.

Therefore, this study constitutes the first approach regarding dysregulation of THs and placental DIO in the placenta from GDM. Another TH regulator involved in placenta, the Thyroid Hormone Transporters (THT), were not observed in this study, and will require further analysis. Therefore, the next goal is to understand how and why these changes happen are to observe placental THT in GDM mothers, to have the full picture behind this alteration.

## Supporting information

S1 File (PPTX)

S1 Data (XLSX)
