# Supplementary material for: High levels of maternal total tri-iodothyronine, and low levels of fetal free L-thyroxine and total tri-iodothyronine, are associated with altered deiodinase expression and activity in placenta with gestational diabetes mellitus
Source: PLoS One. 2020 Nov 24;15(11):e0242743. doi: 10.1371/journal.pone.0242743 (PMC7685482; doi:10.1371/journal.pone.0242743)
Supplement: S1 File — (PPTX) [file pone.0242743.s001.pptx]

## Slide 1
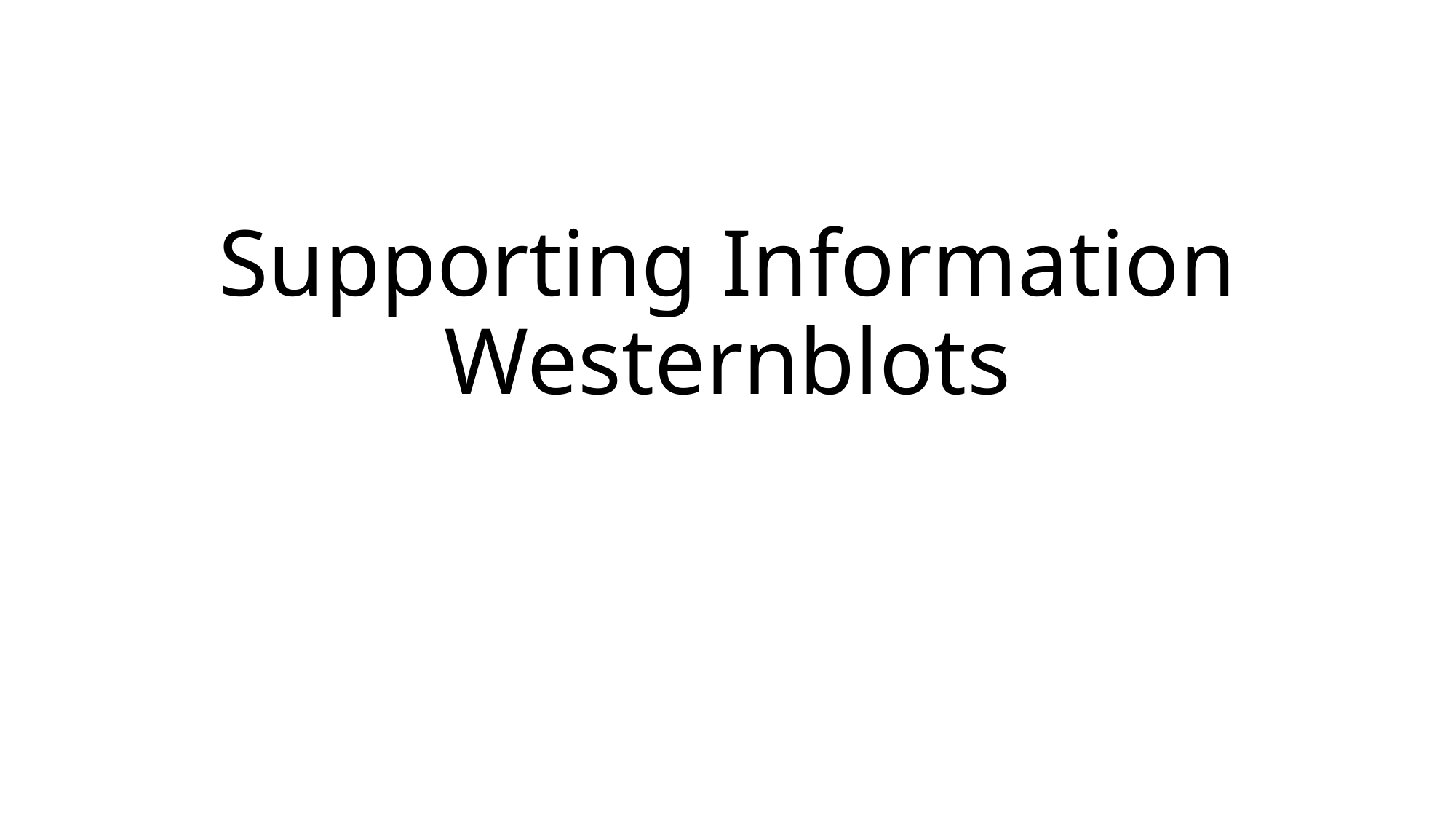

# Supporting Information Westernblots

## Slide 2
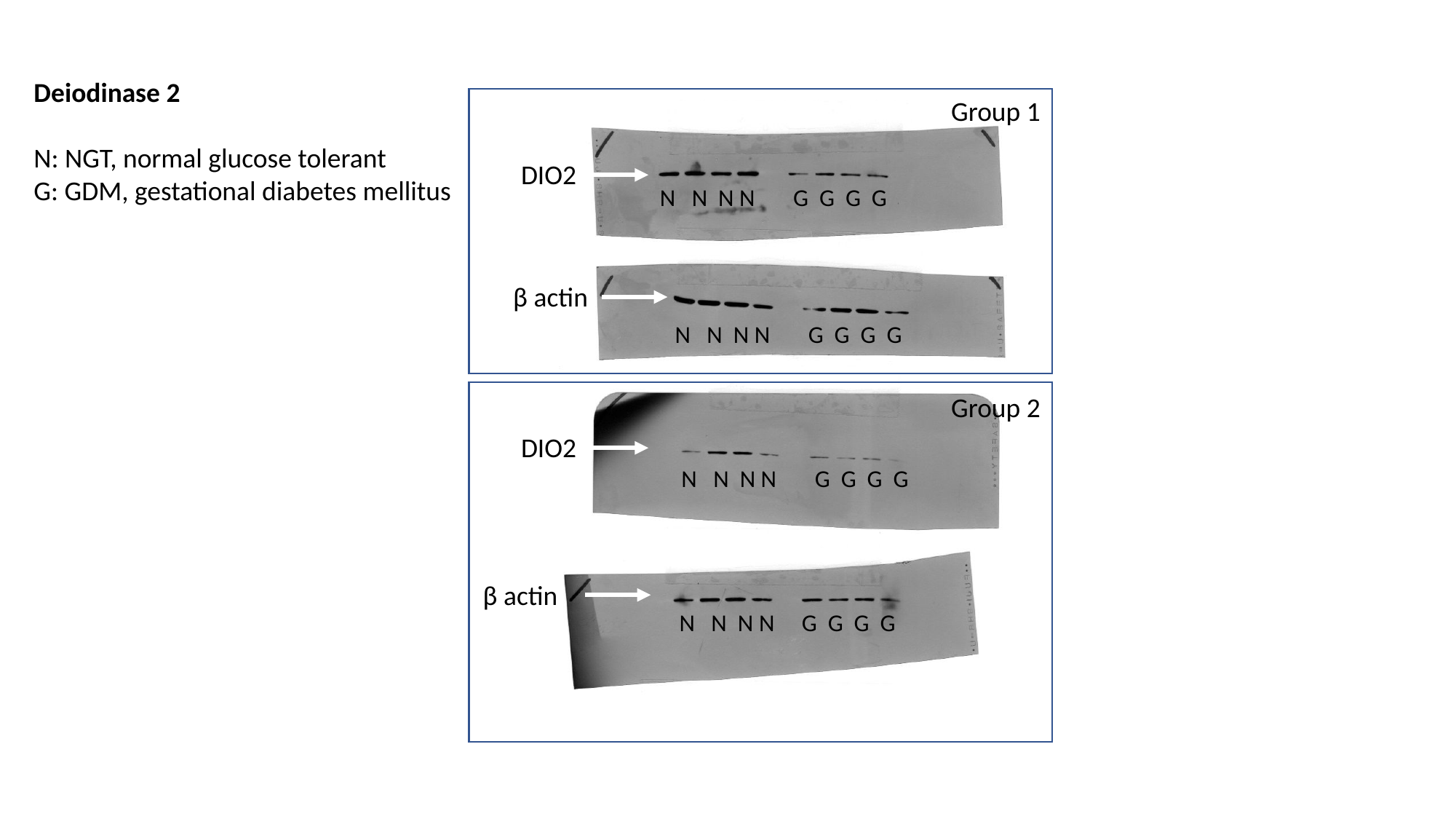

Deiodinase 2
N: NGT, normal glucose tolerant
G: GDM, gestational diabetes mellitus
Group 1
DIO2
 N N N N G G G G
β actin
 N N N N G G G G
Group 2
DIO2
 N N N N G G G G
β actin
 N N N N G G G G

## Slide 3
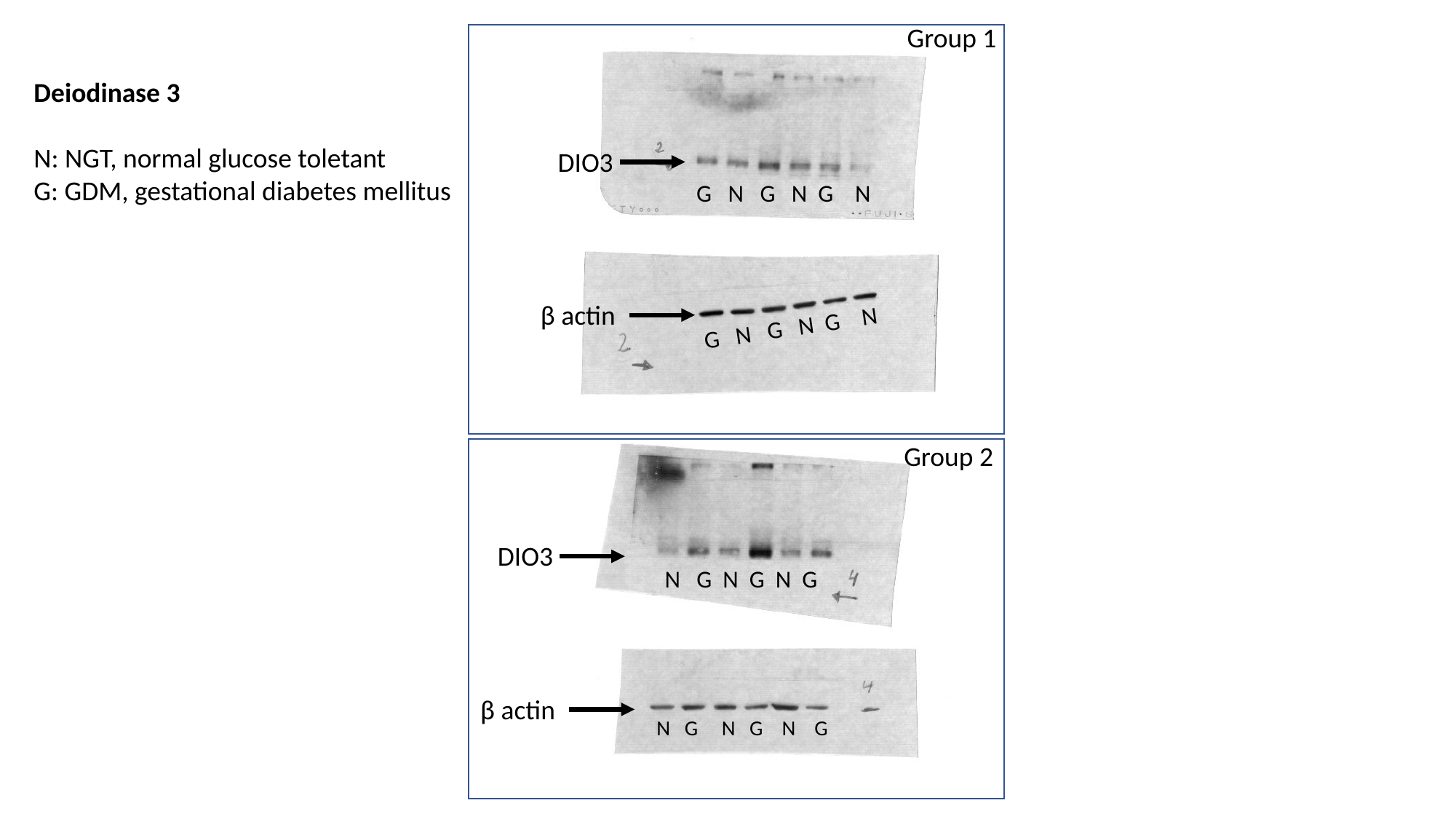

Group 1
Deiodinase 3
N: NGT, normal glucose toletant
G: GDM, gestational diabetes mellitus
DIO3
 G N G N G N
β actin
 G N G N G N
Group 2
DIO3
N G N G N G
β actin
N G N G N G

## Slide 4
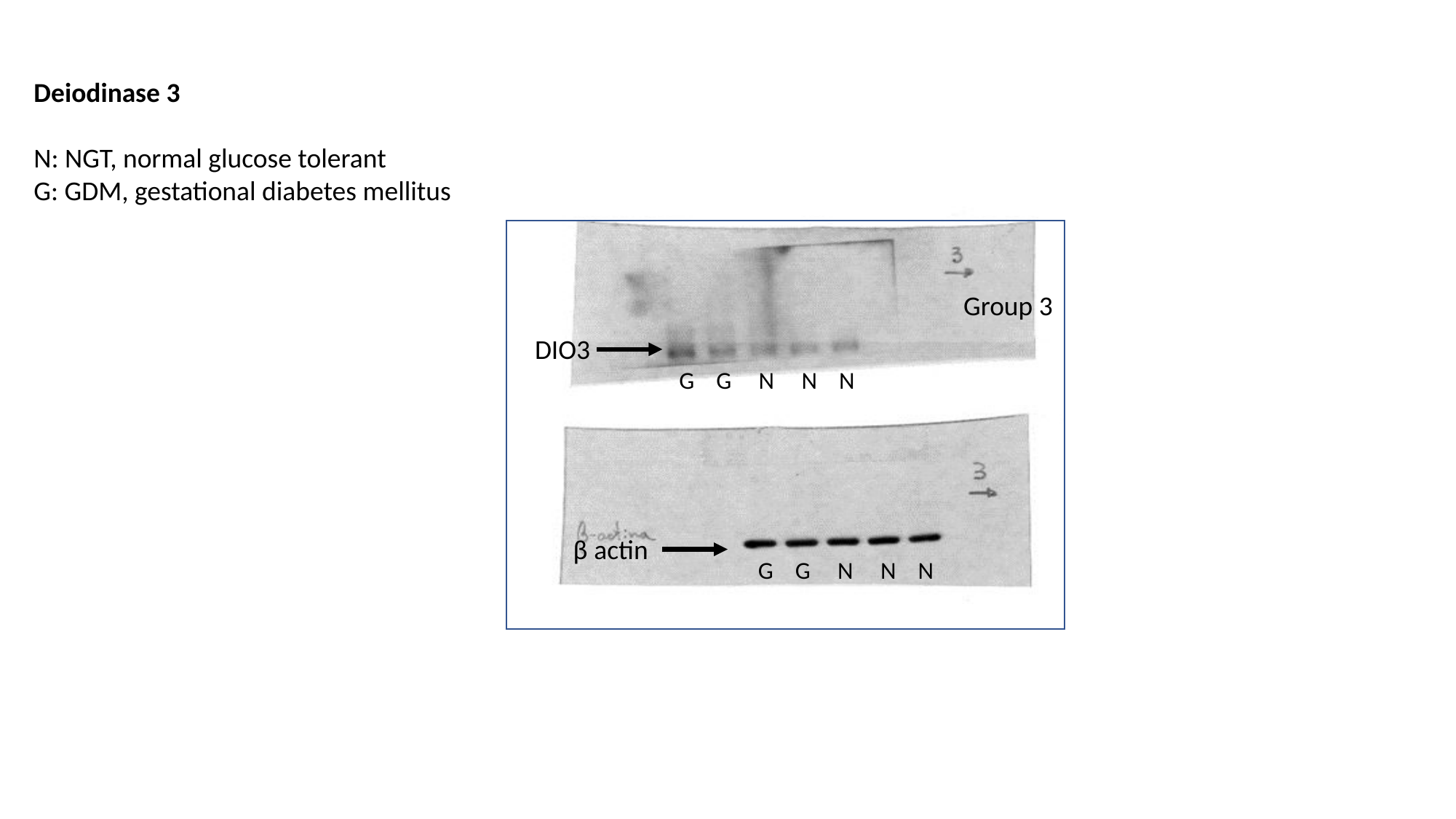

Deiodinase 3
N: NGT, normal glucose tolerant
G: GDM, gestational diabetes mellitus
Group 3
DIO3
 G G N N N
β actin
 G G N N N
